# Development of mAb-based polyglutamine-dependent and polyglutamine length-independent huntingtin quantification assays with cross-site validation

**DOI:** 10.1371/journal.pone.0266812

**Published:** 2022-04-08

**Authors:** David F. Fischer, Sipke Dijkstra, Kimberly Lo, Johnny Suijker, Ana C. P. Correia, Patricia Naud, Martin Poirier, Michela A. Tessari, Ivette Boogaard, Geraldine Flynn, Mijke Visser, Marieke B. A. C. Lamers, George McAllister, Ignacio Munoz-Sanjuan, Douglas Macdonald

**Affiliations:** 1 Charles River, Chesterford Research Park, Saffron Walden, United Kingdom; 2 Charles River, Leiden, The Netherlands; 3 Charles River, Shrewsbury, MA, United States of America; 4 Galapagos, Leiden, The Netherlands; 5 CHDI Management/CHDI Foundation, Los Angeles, CA, United States of America; Rijksuniversiteit Groningen, NETHERLANDS

## Abstract

Huntington’s disease (HD) is caused by an expansion of the CAG trinucleotide repeat domain in the *huntingtin* gene that results in expression of a mutant huntingtin protein (mHTT) containing an expanded polyglutamine tract in the amino terminus. A number of therapeutic approaches that aim to reduce mHTT expression either locally in the CNS or systemically are in clinical development. We have previously described sensitive and selective assays that measure human HTT proteins either in a polyglutamine-independent (detecting both mutant expanded and non-expanded proteins) or in a polyglutamine length-dependent manner (detecting the disease-causing polyglutamine repeats) on the electrochemiluminescence Meso Scale Discovery detection platform. These original assays relied upon polyclonal antibodies. To ensure an accessible and sustainable resource for the HD field, we developed similar assays employing monoclonal antibodies. We demonstrate that these assays have equivalent sensitivity compared to our previous assays through the evaluation of cellular and animal model systems, as well as HD patient biosamples. We also demonstrate cross-site validation of these assays, allowing direct comparison of studies performed in geographically distinct laboratories.

## Introduction

Huntington’s disease (HD) is an autosomal dominant neurodegenerative disorder caused by an expansion of a CAG trinucleotide repeat in exon 1 of the *huntingtin* (*HTT*) gene [1]. The mutant form of HTT containing the polyglutamine expansion is considered cytotoxic and leads to the hallmark pathology of HD, intracellular aggregated proteins [2, 3] and pronounced atrophy of the striatum and other brain regions [4]. Currently multiple investigators have published their progress on developing therapeutic approaches that directly suppress *HTT* expression through RNA interference, antisense technologies, or transcriptional repression [5–12] with some programs already at clinical trial stages [13, 14]. The most recent clinical pharmacodynamic data (NCT02519036) from the non-allele-selective antisense oligonucleotide RG6042 (aka tominersen), showed a prolonged dose-dependent lowering of the mutant huntingtin protein (mHTT) in the CSF of trial participants [15].

Previously, we have characterized several anti-HTT polyclonal antibodies and used them to develop detection assays for human HTT proteins in both a polyglutamine-independent and -dependent (polyglutamine expanded) manner, as well as an assay specific for rodent HTT using the ELISA-based Meso Scale Discovery (MSD) electrochemiluminescence assay platform [16] The advantages of this technology include high selectivity and sensitivity due to the use of a capture and detection antibody which affords an increased dynamic range over several other methods with the ability to multiplex these assays [17]. The MSD platform can be run in high throughput (up to 384-well), with minimal handling/washing steps, and it is an established platform to which many laboratories have access.

**Fig 1:**
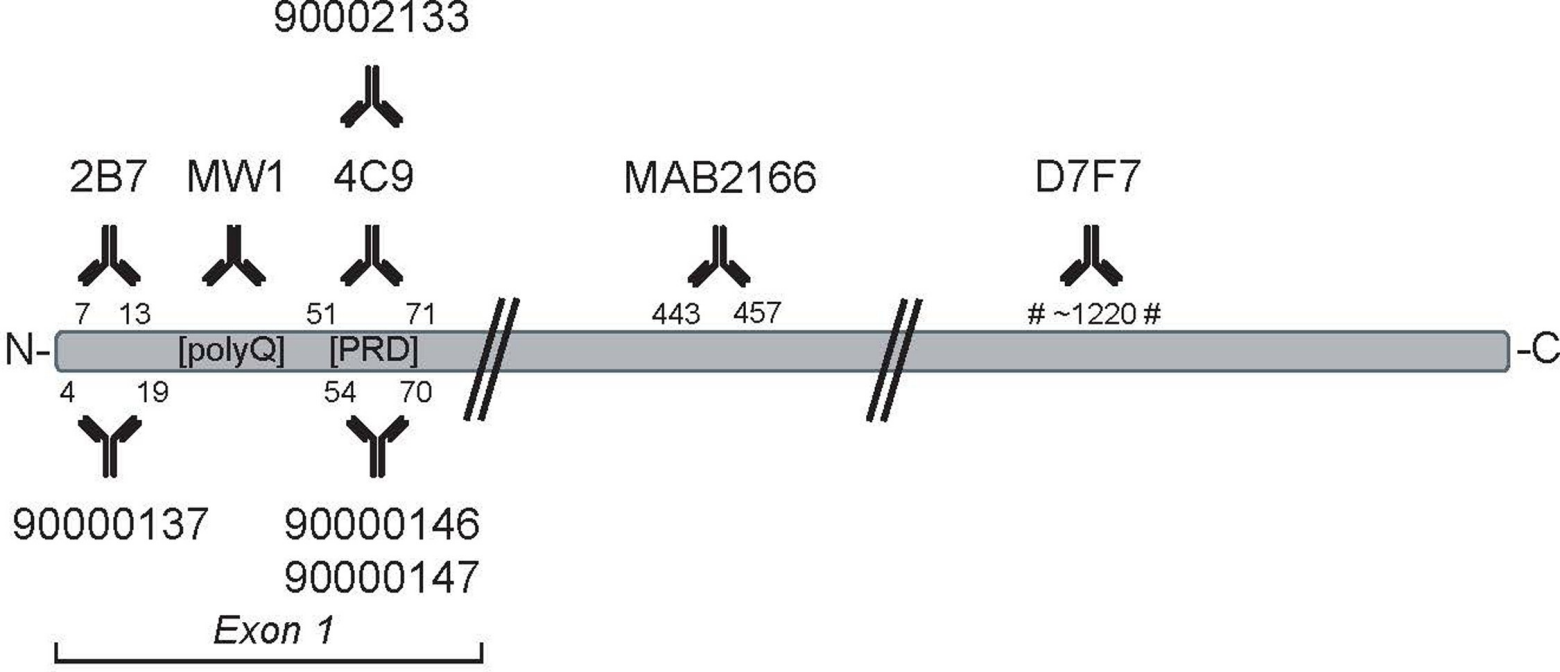
Schematic illustrating the epitopes of anti-HTT antibodies used for MSD assays. Monoclonal and polyclonal antibodies are shown at the top and the bottom, respectively. Antibodies 90000147 and 90002133 are mouse-selective; antibodies 90000146 and 4C9 (when used as a detection antibody) are human-selective, all other antibodies are not species-selective.

Studies using the polyclonal antibody-based assays [16] have now been reported [18–20]. These original assays used at least one rabbit polyclonal antibody, limiting the long-term use of these assays. Several monoclonal antibodies (mAbs) directed against HTT have been developed by a number of parties including Novartis (2B7 [21] and 4C9 [22]), Merck-Millipore (MAB2166-4C8 [23]), CST (D7F7/mAb #5656), as well as CHDI Foundation (CHDI-90002133). Therefore, we sought to replace the polyclonal antibodies with different combinations of these anti-HTT mAbs (Fig 1) to development of HTT quantification assays to add to our platform of assays.

We compared these mAb-based assays to the original assays reported in 2014 [16] using different types of pre-clinical biosamples. We show here that these assays perform at least equivalently to, and sometimes surpass, our original polyclonal-antibody assays in terms of lower-limit of detection, linearity, accuracy, and signal to background. Additionally, since both preclinical and clinical studies typically run across several research sites and sample shipping can cause delays, we successfully cross-validated the assays for HTT in two laboratories, one in Europe and one in the United States. The use of 2B7/4C9 and 2B7/MW1 assays have recently been published by us and other groups [12, 24]. In addition, an MSD assay for aggregated HTT has also been reported [25]. These mAb-based assays are now readily accessible to the wider HD research community (www.chdifoundation.org/research-tools-reagents/) with the antibodies deposited at the Coriell Institute for Medical Research (https://www.coriell.org/).

## Materials and methods

### Recombinant human and mouse huntingtin proteins

#### Purification of large fragment HTT recombinant proteins from Sf9 cells

The human HTT-Q23, -Q45, -Q73 (1–573) and mouse HTT-Q7 (1–549) recombinant proteins were generated as previously described [16]. Briefly, expression was performed using an in-house developed baculovirus–Sf9 insect cell expression system. Proteins were purified by anti-FLAG® M2 affinity chromatography followed by Superdex™ 200 size exclusion in 50 mM Tris-HCl pH 7.4, 500 mM NaCl, 10% glycerol, 0.1% CHAPS and 1 mM EDTA. Since the purity of the mouse HTT protein was not improved following size exclusion chromatography an ion exchange Mono Q chromatography step was included. HTT protein concentration was determined by Bradford assay and purified HTT proteins were stored at -80˚C in size exclusion buffer (S1 Fig). An overview of the different recombinant HTT proteins used in this study is shown in S1 Table.

#### Purification of full-length human HTT proteins from HEK293 cells

The full-length human HTT proteins HTT-Q17 and HTT-Q46 (1–3144) were expressed in HEK293 cells at Charles River CRP. Cell pellets were lysed by one freeze/thaw cycle in 50 mM Tris, pH 8.0, 500 mM NaCl, 5 mM EDTA and protease inhibitors (Complete, EDTA-free; Roche Diagnostics, Cat. 4693159001). Clarification was carried out by centrifugation (61,236 ×g, 2 hours, 4°C) and the soluble lysate was incubated with anti-FLAG® M2 affinity gel (Sigma, Cat. A2220) for two hours at 4°C on a rotator. The resin was column wise washed thoroughly with 5 column volumes of buffer A (50 mM Tris, 500 mM NaCl, 5% v/v glycerol, 0.01% Tween®-20, pH 8.0), then 5 column volumes of buffer B (50 mM Tris, 500 mM KCl, 5mM MgCl_2_, 5% v/v glycerol, 0.01% v/v Tween®-20, pH 8.0). For the HTT-Q17 (1–1344) protein an extra wash step was included using 5 column volumes of buffer C (20 mM Tris, 200 mM KCl, 5 mM MgCl_2_, 5 mM ATP, 0.01% v/v Tween®-20, 5% v/v glycerol, pH 8.0) to reduce HSP70 contamination. Before elution the column was washed with 2 column volumes of buffer A (50 mM Tris, 500 mM NaCl, 5% v/v glycerol, 0.01% v/v Tween, pH 8.0). FLAG-tagged HTT proteins were eluted with 0.1 mg/ml FLAG® peptide in buffer A. Before size exclusion 0.4% CHAPS and 5 mM DTT was added and the proteins were loaded onto a Sephacryl™ S-400 16/60 column equilibrated in 20 mM Tris pH 8, 500 mM NaCl, 5% glycerol, 0.4% CHAPS and 5 mM DTT. Purified HTT proteins were stored at -80˚C at 0.5 mg/mL in the size exclusion buffer (S2 Fig).

Full-length mouse HTT protein HTT-Q7 (1–3120) was expressed in a stable HEK293 in Expi293 expression system by Albany Molecular Research Inc. (Albany, USA), using a cloning vector pcDNA3.1 and restriction enzymes *Nhe*I and *Pme*I. C-terminal FLAG tagged recombinant proteins were purified by FLAG affinity chromatography followed by size exclusion, and equilibrated in 50 mM Tris pH 8.0, 500mM NaCL, 0.5% CHAPS, 1 mM TCEP and 5% glycerol. Purified proteins were stored at -80˚C at 1.0 mg/mL in the size exclusion buffer. An overview of the different recombinant HTT proteins used in this study is shown in S1 Table.

#### Anti-HTT antibodies

Rabbit polyclonal antibody CHDI-90000137 (pAb137) was described previously [16] and recognizes the N-terminus of human and mouse HTT. Rabbit polyclonal antibodies CHDI-90000146 (pAb146) and CHDI-90000147 (pAb147) were generated using antigenic peptides containing the proline-rich region of human and mouse HTT, respectively, as previously described [16]. Rabbit monoclonal antibody CHDI-90002133 (mAb2133) was generated by Thermo Fisher using the ABfinity^TM^ platform with rabbits immunized with the same antigenic peptide used previously for pAb147 (acetyl-pppQPPQPPPQGQPPPPC-amide). Please see S3 Fig for western blot data using this antibody. All CHDI antibodies were provided as purified IgG.

Mouse monoclonal MW1 antibody binding the expanded polyglutamine domain [26] was obtained as hybridoma supernatant from the Developmental Studies Hybridoma Bank and as purified IgG custom produced by Pierce/Thermo Fisher. Mouse monoclonal antibody MAB2166 (clone 1HU-4C8) was obtained as ascites from Merck-Millipore; its epitope has been mapped to amino acids 445–459 of the human HTT protein [27]. Mouse monoclonal antibody 2B7 [21] was raised against the N-terminal domain of human and mouse HTT, whereas 4C9 was raised against the proline-rich region of human HTT (amino acid 65–84; [28]) and was custom produced by Pierce/Thermo Fisher. D7F7 (Cell Signaling Technology mAb #5656) is a recombinant rabbit monoclonal antibody raised using an antigenic peptide corresponding to a sequence surrounding residue 1220 of human HTT.

#### Antibody biotinylation

Biotinylation of antibodies pAb137, MW1 and 4C9 was performed using EZ-Link^TM^ Sulfo-NHS-LC-Biotin (Thermo Fisher, Cat. 21327) according to the manufacturer’s instructions. Briefly, after removing components possibly interfering with biotinylation (e.g., sodium azide, glycerol) with Zeba^TM^ Spin Desalting Columns (40 kDa molecular weight cut-off; Thermo Fisher, Cat. 87766), antibodies were collected in PBS (pH 8.0) and protein concentration was determined by measuring the absorbance at 280 nm using a SimpliNano microvolume spectrophotometer (GE Healthcare). The biotinylation reaction was set up at a 10:1 molar ratio (biotinylating reagent: antibody, based on a MW of 150 kDa for IgG) and incubated at room temperature for 3h with gentle shaking. After labeling, biotin conjugated antibodies were purified in Zeba^TM^ Spin Desalting Columns and stored aliquoted at -20˚C with 30% v/v glycerol. Biotinylated D7F7 antibody was obtained from Cell Signaling Technology.

#### Buffers for tissue lysis and MSD immunoassays

Tissues were lysed using MSD lysis buffer 1 consisting of MSD Tris lysis buffer (Meso Scale Discovery, Cat. R60TX) supplemented with Phosphatase inhibitor II (Sigma, Cat. P5726) and Phosphatase inhibitor III (Sigma, Cat. P0044), 2 mM PMSF (Sigma, Cat. P7626-25G), Complete ULTRA EDTA-free protease inhibitor tablets (Roche Diagnostics, Cat. 5892791001) and 10 mM NaF (Sigma, Cat. S-7920). Samples consisting of cell pellets were lysed using MSD lysis buffer 3 consisting of 1 mM PMSF, 1 mM EDTA, 0,05% SDS and protease inhibitors (Complete, EDTA-free; Roche Diagnostics, Cat. 4693159001) in wash buffer (see below). For coating the MSD plates, antibodies were diluted in carbonate-bicarbonate buffer (15 mM Na2CO3/35 mM NaHCO3, pH 9.6). Washing steps were performed in wash buffer consisting of 0.2% Tween-20 in PBS, while blocking buffer (2% Probumin® BSA (Merck, Cat. 820452) diluted in wash buffer) was used to block non-specific binding to the plates and to dilute the antibodies.

#### Huntington’s disease mouse models

Snap-frozen brain tissue samples from R6/2 B6CBA-Tg(HDexon1)62 Gpb/1 J transgenic mice (CHDI-81001000: JAX strain 002810; CAG~120) and from zQ175 knock-in mice (CHDI-81003003; JAX strain 370437) were provided by the CHDI Foundation from their colony at The Jackson Laboratories (Bar Harbor, USA). Results were compared to brain tissues of three wildtype C57BL/6J female mice (Harlan, USA). Brain tissue from an aged series (12, 24, 36 and 54 weeks) of wild type and Q175DN mice on a C57BL/6J background, which are derived from the original zQ175 mouse model [29–31] by excising the neomycin cassette resulting in zQ175DN mice [32, 33], were provided by the CHDI Foundation from their colony (CHDI-81003019 / JAX strain 370832 for heterozygous zQ175DN and JAX strain 400823 for homozygous zQ175DN mice; CAG ~180–220) at the Jackson Laboratories (Bar Harbor, ME, USA). Mouse tissue samples were provided with neither intervention nor treatment of animals.

#### Tissue homogenization

Tissue homogenization was performed as previously described [16]. Briefly, dissected or whole brain tissues were homogenized and lysed in MSD lysis buffer 1 using the FastPrep 24 homogenizer (MP Biomedicals) for 3x30 s in Lysing matrix tubes (Lysing matrix D; MP Biomedicals, Cat. 116913500). Volume of lysis buffer was based on the average weight of the tissue, i.e. <10 mg add 200 μl, 10–50 mg add 250 μl and >50 mg add 300–400 μl lysis buffer. Afterwards, lysates were centrifuged 2 x 20 min at 20,000 ×g at 4˚C and supernatant was collected, aliquoted and frozen on dry ice. Lysates were stored at -80˚C for a maximum of 3–4 days until analysis.

Total protein concentrations were determined by bicinchoninic acid assay (BCA; Thermo Scientific, Cat. 23225) according to the manufacturer’s instructions. Briefly, three different sample dilutions were prepared, and each measured in triplicate to ensure that samples fall within the linear range of the BSA reference curve (typically 250–1500 μg/ml). For each sample dilution, 97.5 μl of working reagent was added to 2.5 μl sample dilution and incubated for 30 minutes at 37˚C, followed by measuring the absorbance at 535 nm on an Envision plate reader (PerkinElmer).

#### MesoScale discovery platform assays

MSD assays for detection and quantitation of various HTT protein species were performed as previously described [16]. Briefly, 96-well MSD plates (Meso Scale Discovery) were coated overnight at 4°C with the pAb146 polyclonal antibody (8 μg/ml) or the monoclonal antibody 2B7 (1.5 μg/ml) for ‘polyclonal’ and ‘monoclonal’ versions, respectively, of assays detecting polyglutamine-expanded HTT or polyglutamine independent human HTT assays. For the detection of mouse HTT, plates were coated with the polyclonal antibody pAb147 (4 μg/ml) or the monoclonal antibody mAb2133 (8 μg/ml). Plates were washed three times with wash buffer and blocked with blocking buffer for 1h at RT with rotational shaking. Samples were protein normalized in the corresponding lysis buffer and diluted 5 times in blocking buffer to reduce possible interference from lysis buffer components. A volume of 25 μl of the diluted samples was transferred to the MSD plate and incubated for 1h at RT with rotational shaking. After washing 3 times with wash buffer, 25 μl of detection antibodies was added, diluted in block buffer. Hybridoma supernatant MW1 (1.0 μg/ml) or biotinylated purified MW1 (0.625 μg/ml) was added for expanded polyglutamine HTT assays, and biotinylated pAb137 (2.5 μg/ml) or biotinylated 4C9 (0.5 μg/ml) was added for polyglutamine independent HTT assays. For the mouse HTT assays, 25 μl of the monoclonal antibody MAB2166 (1:10,000 dilution) or biotinylated D7F7 (1.5 μg/ml) was used as first detection step. After 1h incubation at RT with rotational shaking, plates were washed 3 times and 25 μl of the secondary detection reagents was added, diluted in blocking buffer. Goat anti-mouse SULFO-TAG detection antibody (1: 2,000; Meso Scale Discovery, Cat. R32AC) was used for assays with an unlabeled mouse antibody as first detection step, while streptavidin-SULFO-TAG (diluted 1: 10,000; Meso Scale Discovery, Cat. R32AD) was used for assays with biotinylated antibodies as first detection step. Secondary detection reagents were incubated with shaking for 1h at RT. After washing, 150 μl read buffer (Meso Scale Discovery, Cat. R92TC) was added to each empty well and the plate was imaged on a MESO SECTOR S 600 imager (Meso Scale Discovery) according to manufacturers’ instructions and settings recommended for 96-well plates.

#### Data analysis

Where applicable, HTT concentrations were calculated using a standard curve of the appropriate recombinant protein. Interpolation of instrument responses was performed using the ‘Discovery workbench’ software (Meso Scale Discovery). Concentrations were only calculated for samples with MSD signals within the linear part of the standard curve. The percentage coefficient of variation (%CV) was calculated using the average and standard deviation of technical replicates each sample. Average %CV was typically <15%.

#### Statistical analysis

Unless otherwise indicated, the overlays in the graphs represent averages with standard errors of the mean. Statistical significance of differences between groups was calculated using one-way ANOVA with Dunnett’s post hoc test, unless otherwise indicated.

## Results

### Assay comparison with recombinant HTT proteins

We previously reported MSD immunoassays for the detection and quantification of mutant polyglutamine-expanded human HTT recognizing HTT proteins with a polyglutamine repeat length ranging from Q40 to Q73 utilizing the antibody pair of pAb137/MW1 (capture and detection, respectively) and for detection and quantification of polyglutamine length independent human HTT (non-expanded and polyglutamine-expanded HTT) utilizing the antibody pair of pAb146/pAb137 [16]. Here we replaced the polyclonal antibodies in those assays with mAbs recognizing similar domains of the HTT protein (i.e., 2B7 for pAb137 (HTT N-terminus) and 4C9 for pAb146 (proline rich domain of human HTT). In addition, we reported an MSD assay for monitoring endogenous HTT levels in rodent HD models (pAb147/MAB2166) [16], for which we have now also developed an alternative assay using only monoclonal antibodies (mAb CHDI-90002133/D7F7). The antibody D7F7 provided higher sensitivity compared to MAB2166, and moreover, it has been reported that MAB2166 binding is sensitive to phosphorylation [34]. Fig 1 illustrates the epitopes of the various antibodies used in this study.

**Fig 2:**
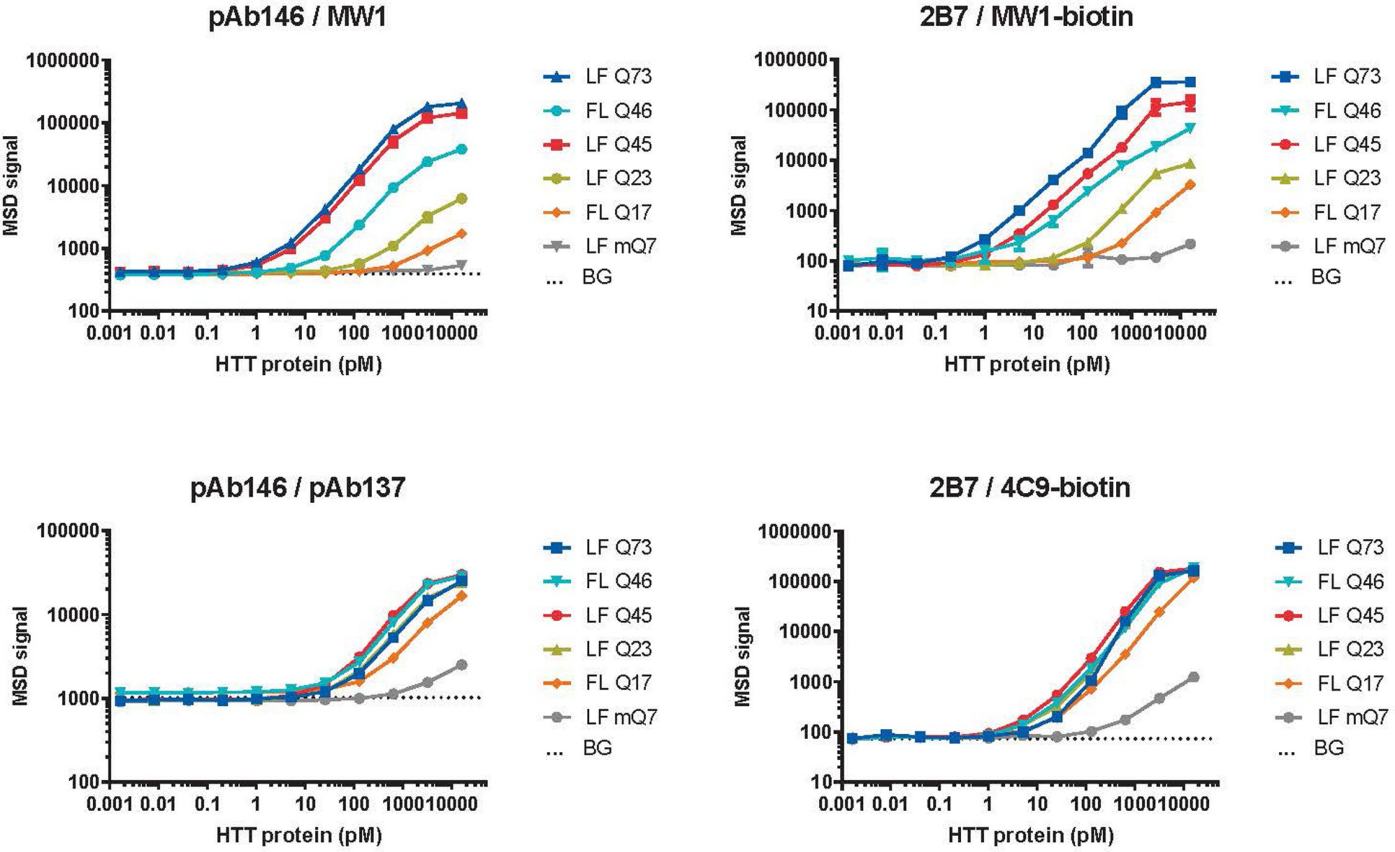
Enhanced sensitivity and dynamic range in HTT MSD assays employing only monoclonal antibodies compared to assays using polyclonal antibodies. Recombinant human HTT proteins of different fragment length (1–573 or full length) and Q length, and mouse HTT-Q7 (1–549) large fragment (LF) were spiked into MSD lysis buffer 1 and analyzed with expanded polyglutamine HTT assays employing antibody pairs pAb146/MW1 (‘polyclonal assay’) or 2B7/MW1 (‘monoclonal assay’), or with exon-1 pan-human HTT assays employing antibody pairs pAb146/pAb137 or 2B7/4C9. Mean ± s.d. of n = 4 technical replicates obtained from two different MSD plates. BG, background signal obtained from MSD lysis buffer 1. Horizontal dotted lines indicate LLoD calculated from BG + 3 standard deviations using the LFQ73 curve. LF, large N-terminal fragment (N573) of human HTT; FL, full length human HTT. Please note that antibodies are shown in the order of capture / detection. Please note that the error bars are smaller than the symbol used in the graph and therefore cannot be seen.

Optimization experiments testing various antibody concentrations, combinations and orientations (not shown) resulted in ‘monoclonal’ MSD assays for polyglutamine expanded HTT (capture with 1.5 μg/ml 2B7, detection with 0.625 μg/ml biotinylated MW1), for polyglutamine length-independent human HTT (capture with 1.5 μg/ml 2B7, detection with 0.5 μg/ml biotinylated 4C9) and for rodent HTT (capture with 8 μg/ml mAb2133, detection with 1.5 μg/ml biotinylated D7F7, both rabbit monoclonal antibodies). As shown in Fig 2, validation experiments with assay 2B7/MW1 titrating serially diluted recombinant HTT protein fragments (amino acids 1–573) of different polyglutamine repeat lengths (Q23, Q45 or Q73) confirmed polyglutamine length dependency of the signals as expected. Similar polyglutamine dependency of the signal was observed with full-length human HTT proteins. Compared to its polyclonal counterpart (pAb137/MW1), assay 2B7/MW1 showed a slightly enhanced sensitivity and dynamic range (Table 1).

**Table 1:** Sensitivity of HTT immunoassays compared to existing described in this paper. Recombinant human HTT-Q73 (1–573) or mouse full-length HTT-Q7 was spiked into lysis buffer or mouse brain lysate (derived from wild type or homozygous zQ175DN mice for spiking human or mouse recombinant HTT, respectively). Serially dilutions of recombinant HTT in each of the matrices were measured with the indicated HTT MSD immunoassays (indicated as antibody pairs: capture/ detection), and LLoD concentration was back-calculated based on mean background signal + 3 x standard deviation. Dynamic range was estimated visually from the standard curves, which are shown in S4 Fig. *determined in 3 and 10 μg brain lysate instead of 20 and 40 μg, respectively.

	Matrix	Expanded HTT assays (hQ73)		Total human HTT assays (hQ73)		Mouse HTT assays (mQ7)	
		pAb146/ MW1	2B7/ MW1-bio	pAb146/ pAb137-bio	2B7/ 4C9-bio	pAb147/ MAB2166	mAb2133/ D7F7
	Assay number	CHDI_HTT_001	CHDI_HTT_006	CHDI_HTT_002a	CHDI_HTT_039	CHDI_HTT_003	CHDI_HTT_141
**LLoD**	Lysis buffer	~ 0.6 pM	~ 0.3 pM	~ 20 pM	~ 6 pM	0.3 pM	0.4 pM
	20 μg brain lysate	~ 2 pM	~ 1 pM	~ 50 pM	~ 6 pM	0.5 pM*	0.4 pM*
	40 μg brain lysate	~ 4 pM	~ 1 pM	~ 50 pM	~ 8 pM	0.5 pM*	0.7 pM*
**Dynamic range (Log units)**	Lysis buffer	~2	~3	>1.5	~3	~ 2	~ 2
	20 μg brain lysate	~2	~3	>1.5	~2.5	~ 2*	~ 2*
	40 μg brain lysate	~2	~3	>1.5	~2.5	~ 2*	~ 2*

Assay 2B7/4C9, designed to recognize human HTT in a polyglutamine length-independent manner, showed similar signals across recombinant HTT proteins of different polyglutamine lengths, in the context both of a large N-terminal fragment (1–573) and of full-length (FL; 1–3144) HTT protein (Fig 2). Compared to its polyclonal counterpart assay (pAb146/pAb137) both sensitivity and dynamic range were 5–10 fold enhanced (Table 1).

**Fig 3:**
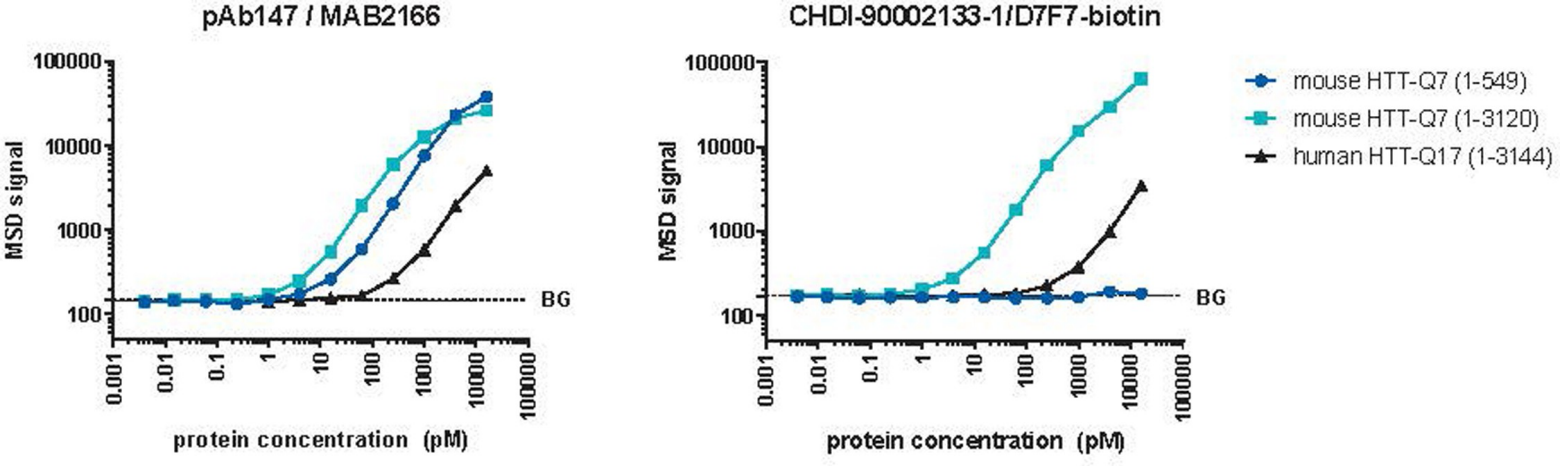
Performance of the mouse HTT MSD assay employing only monoclonal antibodies (mAb2133/D7F7) compared to assays using polyclonal antibodies (pAb147/MAB2166). Recombinant HTT protein curves representing mouse HTT-Q7, 1–549, full-length mouse HTT-Q7 and full-length human HTT-Q17. Please note the logarithmic scales used and that the error bars are smaller than the symbol used in the graph and therefore cannot be seen.

Finally, assay mAb2133/D7F7 recognizing mouse and rat (see supplementary data for sequence comparison of the mAb2133 epitope) endogenous HTT showed similar performance with full length mouse recombinant HTT proteins as the pAb147/MAB2166 antibody pair previously developed (Fig 3). As expected, based on the location of the D7F7 epitope (around amino acid 1220 of HTT), no signal was detected with a mouse HTT (1–549) protein fragment.

### Assay comparison with HD mouse model samples

To validate these assays for use in mouse samples, we first performed spike and recovery experiments in mouse brain lysates for both the existing and full monoclonal assays, to establish the LLoD and assess possible matrix inhibition. Recombinant HTT protein was spiked into assay diluent or into different concentrations of wild-type mouse brain homogenate and serially diluted, using human HTT-Q73 (1–573) protein for assay pairs detecting polyglutamine expanded HTT or polyglutamine-independent human HTT, and mouse HTT-Q7 (1–549) protein for assays detecting rodent HTT. Whereas wild type mouse brain lysates were used for spiking of human recombinant HTT, we used homozygous zQ175 DN knock-in mouse brain lysates for spiking mouse recombinant HTT. Mice of this genotype have both endogenous mouse alleles replaced with a chimeric huntingtin allele which has the human proline-rich region and thus its product is recognized by neither the pAb147/MAB2166 nor the mAb2133/D7F7 antibodypair.

As shown in S4 Fig, both the ‘polyclonal’ and ‘monoclonal’ assays for polyglutamine expanded HTT and polyglutamine length-independent human HTT showed approximately two-fold lower MSD signals in wild type mouse brain lysate matrix compared to assay diluent, indicating some matrix inhibition. When comparing these assays with their polyclonal antibody counterparts, the LLoD was similar for expanded polyglutamine HTT assays pAb146/MW1 and 2B7/MW1 (both low pM range in brain lysate) (Table 1). In contrast, the monoclonal polyglutamine length-independent human HTT assay (2B7/4C9) had much improved LLoD compared to assay pAb146/pAb137 (low pM range compared to ~50 pM, respectively). Compared to the polyglutamine-expanded and polyglutamine length-independent human HTT assays, assays detecting mouse HTT showed only little matrix inhibition of mouse full-length HTT-Q7 detection in homozygous zQ175DN mouse brain lysate (which lacks the endogenous mouse HTT exon 1 domain) (S4 Fig). Similar results were observed for both the full monoclonal assay (mAb2133/D7F7) and the one previously [16] reported (pAb147/MAB2166). Concomitantly, the LLoD was similar between assays, around 1 pM (Table 1).

**Fig 4:**
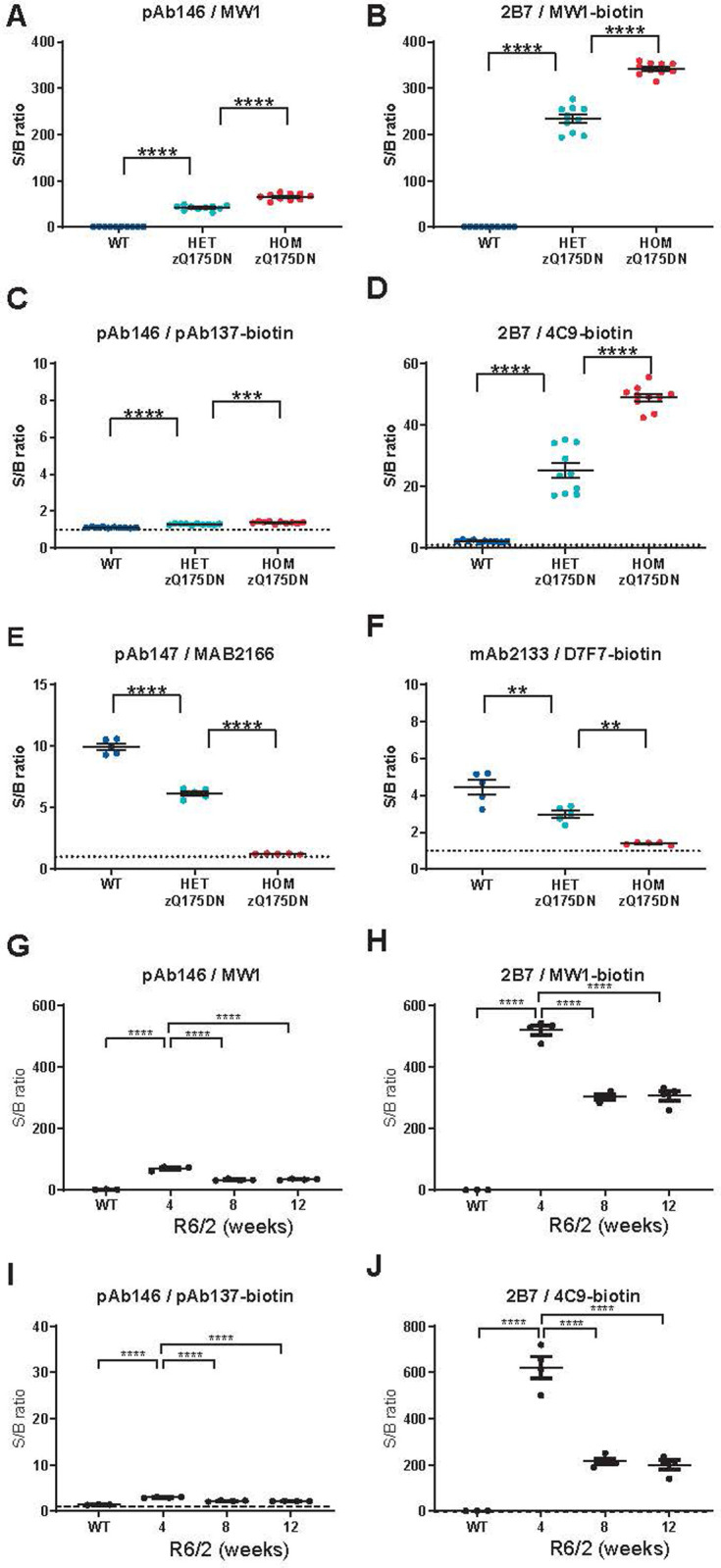
Comparison of HTT MSD assays employing only monoclonal antibodies (right-hand panels) with existing assays using polyclonal antibodies (left-had panels). Panels show gene dosage effect in wild type, heterozygous and homozygous zQ175DN knock-in mouse striatal lysates (A-D) and zQ175DN mouse brain hemisphere lysates (E, F), and age-related HTT levels in wild type and R6/2 mouse brain hemisphere lysates (G-J). Dissected snap-frozen tissues were lysed and analyzed with HTT MSD assays employing the indicated antibody combinations for capture/detection of polyglutamine-expanded HTT (A,B,G,H), total human HTT (C,D,I,J) or mouse HTT (E,F). Each data point shows the average of n = 3 technical replicates; overlays show mean ± SEM by genotype (Q175 n = 10 per group; Q175DN n = 5 per group; R6/2 n = 4 per group). Data are shown as MSD signal over background ratio. Data in A–F were analyzed with unpaired t-tests with Bonferroni correction, data in G-J with Dunnet’s post hoc test following ANOVA. ****p < 0.0001; ***p < 0.001, **p < 0.01.

Next, we compared the performance of the full monoclonal assays and the existing assays in brain tissue lysates from zQ175DN mice (containing the neomycin cassette) and R6/2 mice, both widely used animal models of HD [31, 35]. In striatal lysates prepared from wild type, heterozygous and homozygous zQ175DN mice, both assay pAb146/MW1 and assay 2B7/MW1 showed a good signal over background, as well as an incremental increase in signal with the number of mutant HTT alleles present in heterozygous and homozygous zQ175DN mice (Fig 4A and 4B). Both polyglutamine expanded HTT assays produced minimal signal over buffer background in lysates from wild type striata even at high sample input (25 μg total protein /well tested in this experiment), indicating good specificity for mutant HTT over non-expanded mouse HTT. In heterozygous or homozygous zQ175DN brain samples, assay 2B7/MW1 displayed ~7-fold higher MSD signals than assay pAb146/MW1 showing a better performance. We then evaluated the assay using R6/2 brain samples. We previously described the decrease in soluble HTT protein levels detected in lysates due to the aggregation pathology that occurs during the disease course [16]. As shown in Fig 4G and 4H, pAb146/MW1 and 2B7/MW1 assays both revealed the expected [22] drop in signal representing soluble mutant HTT in brain lysates from 8- and 12-week old compared to 4-week old R6/2 mice.

Assay 2B7/4C9 for the detection of polyglutamine length-independent HTT with a human proline rich domain sequence showed a dramatic improvement in signal over background compared to pAb146/pAb137 using zQ175DN mouse striatal lysates (Fig 4C and 4D) and R6/2 brain lysates (Fig 4I and 4J). Furthermore, the 2B7/4C9 signal was able to detect a 2-fold difference in mHTT levels due to gene dosage in heterozygous compared to homozygous zQ175DN mice, and also able to detect age-dependent changes in mHTT soluble pools in the R6/2 samples. In contrast to assay 2B7/MW1, mouse brain lysates had a higher background signal compared to lysis buffer, which most likely represents mouse HTT, in line with the data presented in Fig 2 on mouse recombinant HTT protein.

The homozygous zQ175DN mouse is an excellent model to test the specificity of immunoassays detecting the mouse HTT protein, as the exon 1 sequence used in the knock-in construct are of human origin in this mouse line. As shown in Fig 4E and 4F), assay mAb2133/D7F7 showed the expected signal decrease with the number of mouse *Htt* alleles present in brain lysates of wild type (n = 2), heterozygous zQ175DN (n = 1) and homozygous zQ175DN (none) mice (Fig 4), and specificity (i.e. gene dosage trend, losing >90% of the signal over background when comparing wild type mice to the homozygous zQ175DN mice, which lack the epitope for mAb2133) was similar between assays, while signal over background ratio was slightly better with the existing assay.

### Age-dependent decrease of mutant human HTT protein levels in Q175DN mouse brain samples

In the previous section we described an age-dependent decrease in mutant HTT levels in R6/2 mouse brains as measured with the pAb146/MW1, pAb146/pAb137, or the 2B7&/MW1 and 2B7/4C9 assays. We also characterized the age-dependent changes in HTT levels in brain lysates of zQ175DN mice [32].

Brains from wild type and heterozygous zQ175DN mice aged 3 to 12 months were isolated, cut into the two hemispheres, homogenized and analyzed with 2B7/MW1 and 2B7/4C9 assays. Endogenous mouse HTT was measured using assay pAb147/MAB2166. Recombinant HTT proteins were used to back-calculate HTT concentrations to allow comparisons between experiments and between laboratories. Please note that as we used a Q73 protein to back-calculate mHTT protein levels from zQ175DN samples carrying an average repeat expansion of 200 glutamines, a significant overestimation of the concentration is made, and we suggest not to use absolute quantification, but to only use a Q73 equivalent value for relative comparison between plates and experiments.

**Fig 5:**
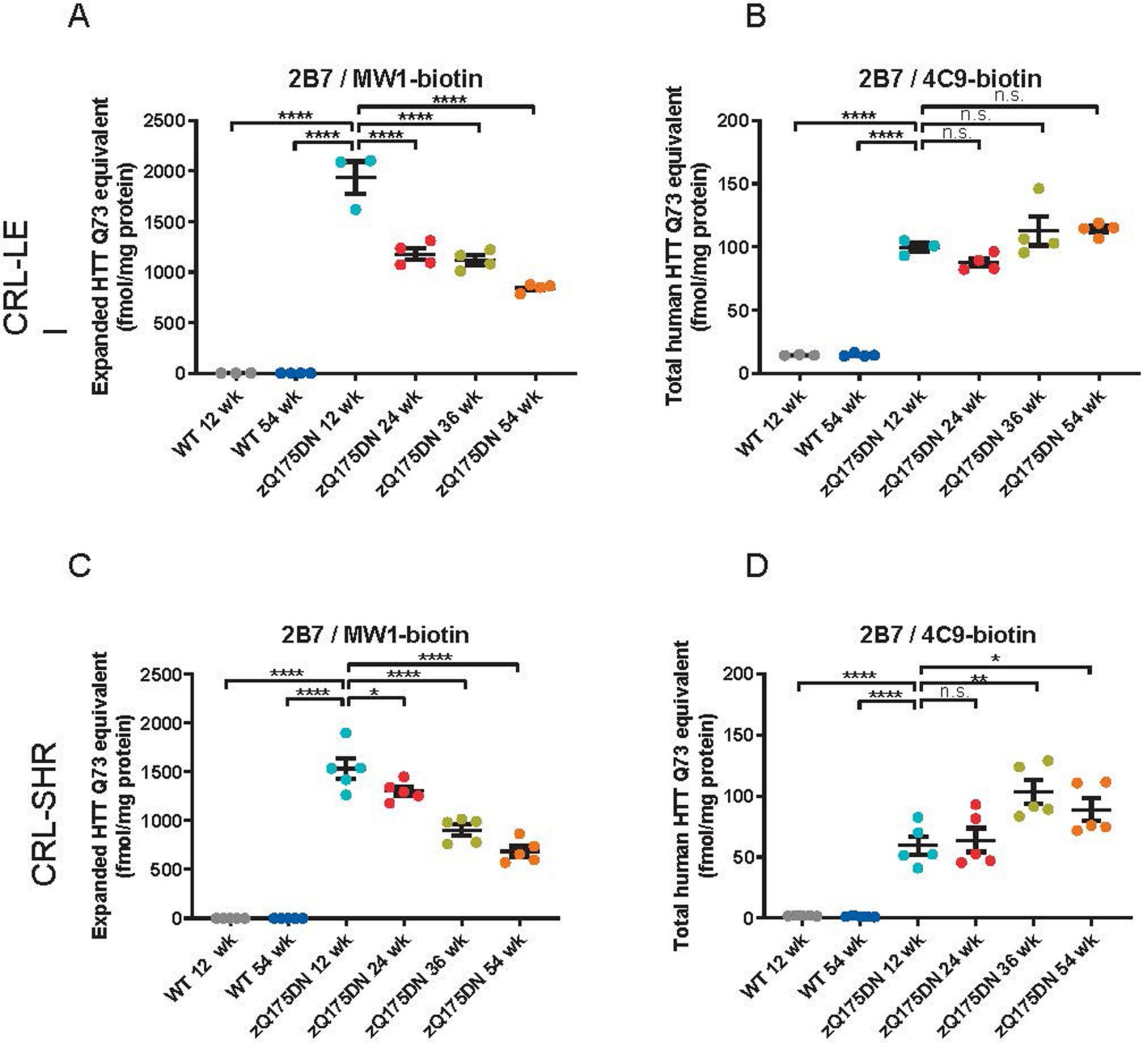
Characterization of HTT protein levels in heterozygous zQ175DN mouse brain over time, as assessed in two independent laboratories in the Netherlands (CRL-LEI, A and B)) and in the U.S. (CRL-SHR, C and D) using the MSD platform. The Q175 allele product was quantitated with two different MSD assays (2B7/MW1 for polyglutamine expanded HTT (A and C) and 2B7/4C9 for total human HTT (B and D)). Please note that the much longer polyglutamine expansion in the sample (zQ175DN has an average of Q200) compared to the reference protein (Q73) leads to an overestimation of the HTT concentration by the assays involving MW1. ****p < 0.0001; ***p < 0.001, **p < 0.01 and *p < 0.05 by Dunnet’s post hoc test following ANOVA.

As shown in Fig 5, mutant HTT levels measured with assay 2B7/MW1 were approximately 1.8 pmol Q73 equivalent/mg total protein in 3 months old zQ175DN HET striata, when back-calculated from a standard curve of recombinant human HTT-Q73 (1–573). In 6 to 9 months-old animals, soluble mutant HTT levels were approximately two-fold lower than in 3-months old ones, whereas after 12 months they had further declined to approximately one third of the level observed in the 3 months group. In contrast, no age-related reduction in the zQ175DN mutant HTT product was observed when the same striatal lysates were analyzed with the polyglutamine length-independent assay 2B7/4C9. In a different cohort of zQ175 (neo containing) mice analyzed previously we had observed an age-related decline reminiscent of the pattern observed with 2B7/MW1 (S7 Fig).

To further test the robustness of the full monoclonal antibody-based assay platform, the assays were then transferred from the first laboratory in Leiden, The Netherlands to a second laboratory in Shrewsbury, Massachusetts, USA, by providing detailed standard assay procedures and reagents. Following reagent and assay transfer experiments with recombinant HTT proteins and mouse brain lysates in the CRL-SHR laboratory, a set of brain samples from a second cohort of zQ175DN mice of similar ages was used to quantitate mutant HTT and mouse HTT protein levels. To this end, snap-frozen brain halves of heterozygous zQ175DN mice and wild type mice aged 3, 6, 9 or 12 months (n = 5 per group), were homogenized and analyzed at the CRL-SHR laboratory with assays 2B7/MW1 and 2B7/4C9 to measure the Q175 allele product. HTT concentrations were back-calculated using standard curves of the most appropriate recombinant HTT proteins. Please note that for assay 2B7/MW1, a Q73 standard protein was used and therefore it cannot be used to quantitate absolute concentrations of the analyte, but the use of the standard curve does however allow for inter-experiment and even inter-laboratory comparison as the Q73 equivalent concentrations were very much in line between the two laboratories with different operators and using a different instrument.

**Fig 6:**
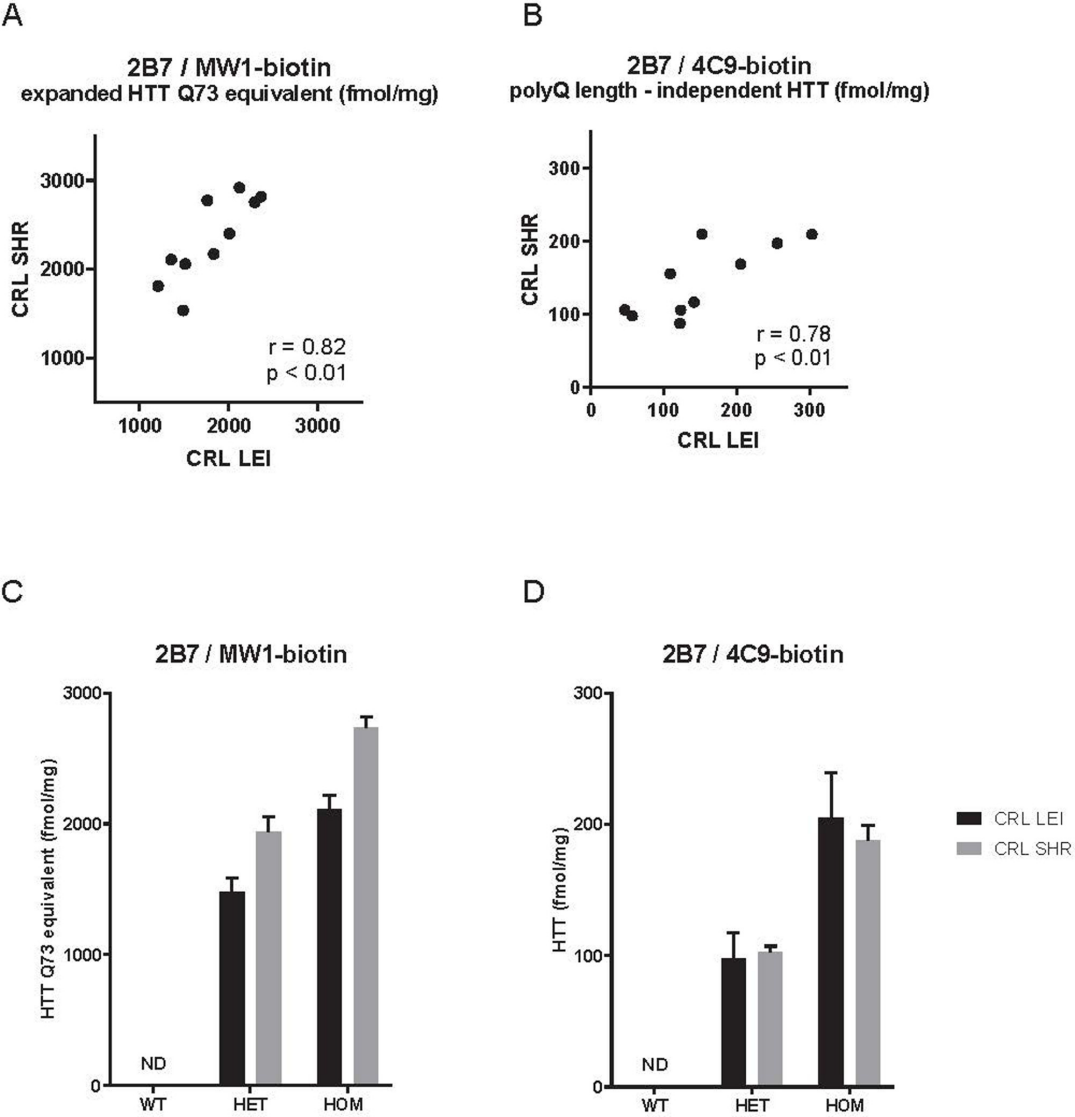
Correlation between HTT protein levels determined in the same lysates of heterozygous zQ175DN mouse brain lysates in two independent laboratories in the Netherlands (CRL-LEI) and in the U.S. (CRL-SHR). The zQ175DN allele product was quantitated with two different MSD assays (2B7/MW1 for polyglutamine expanded HTT (A and C) and 2B7/4C9 for total human HTT (B and D)).

As shown in Fig 5 (bottom panel), similar age-related changes in brain HTT levels were observed in the second cohort zQ175DN HET mice as in the first cohort analyzed in The Netherlands CRL-LEI laboratory (cf. Fig 5 (top panel). Levels of the zQ175DN allele product were ~1.5 pmol Q73 equivalent /mg total protein as measured using assay 2B7/MW1 with a HTT-Q73 (1–573) standard curve, and declined gradually with age to ~700 fmol Q73 equivalent /mg at 12 months. Polyglutamine-independent Human HTT levels measured with 2B7/4C9 did not show this age-dependent reduction. Statistical analysis even suggested an increase in 2B7/4C9 signal with age at 9 and 12 months. A more direct site comparison was also performed by analyzing the same mouse brain lysates at both sites (with overnight shipment on dry ice). Here (Fig 6) a very close match (<30% difference) between individual sample concentrations was seen for two HTT assays. Taken together, these results indicated that even in two separate cohorts of mice from the same strain analyzed in different laboratories with the full monoclonal HTT MSD assays, the results were reproducible.

To analyze assay performance in both laboratories more closely, several performance measures were extracted from the data and compared between laboratories (S5 Fig and Table 2). Standard curves of recombinant HTT proteins were similar in visual appearance for both assays (2B7/MW1, 2B7/4C9, see S5 Fig), resulting in a similar lower limit of detection (LLOD) and dynamic range between laboratories (Table 2). The precision (%CV, coefficient of variation) for technical replicates of the same sample was < 20%, with slightly higher %CV values observed in the US than in NL.

**Table 2:** Comparison of HTT MSD assay performance in two independent laboratories. Performance measures derived from the dataset shown in Fig 5.

Parameter	2B7/4C9-biotin		2B7/MW1-biotin	
	NL (CRL-LEI)	US (CRL-SHR)	NL (CRL-LEI)	US (CRL-SHR)
LOD	~6 pM	1.5 pM	0.1 pM	0.2 pM
Dynamic range	~3.5 logs (6 to >4000 pM)	2–4000 pM	~3.5 logs (0.1–4000 pM)	0.5–4000 pM
Precision	0.3–7% CV	4–20% CV	0.3–10%CV	3–12% CV
Range of HTT concentrations in Q175 WT brains (fmol/mg protein)	37–46	1.1–2.5	<LLOQ	LLOQ-2.7
Range of HTT concentrations in Q175 HET brains (fmol/mg protein)	139–306	41–129	644–1415	572–1899

LOD, limit of detection; LLoQ, lower limit of quantification.

## Discussion

Great strides have been made over the past decade in developing therapeutic approaches for HD [36]. As a number of these strategies aim to directly reduce the expression burden of mHTT in the brain [37], different assay platforms have been developed to measure soluble HTT levels in preclinical [16] and clinical samples [38–40] as a pharmacodynamic readout of HTT lowering. Additional investigations are also underway to develop PET imaging ligands that bind to aggregated HTT in patients to assess HTT levels more directly [36]. Here we have revisited our previously-published assays that can quantify various human, mouse, and polyglutamine expanded forms of HTT protein in pre-clinical models of HD [16] and have replaced the polyclonal antibodies used in the original assays with mAbs. Furthermore, with large scale production of mAb stocks, this expanded assay portfolio will be serviceable for an extended period of time. These full monoclonal assays display greater sensitivity than prior assays, especially when interrogating tissue lysates. When comparing the lower limit of detection, the major advantage of the full mAb assays has been in the analysis of tissue lysates, which can likely be explained by spurious reactivity of a minor fraction of the polyclonal antibody pool that is absent in a mAb. Importantly, this has brought the polyglutamine length-independent human HTT assay (2B7/4C9-bio assay) closer in sensitivity (8 pM) to the mHTT assay (1 pM) compared to the original polyclonal polyglutamine length-independent HTT assay (50 pM), allowing it to be used in parallel with the mHTT assay and the endogenous mouse HTT assay (mAb2133/ D7F7 assay) using similar protein input from tissue samples.

With the development of single molecule counting (SMC)/digital ELISA technologies such as the SMC Erenna and xPro (Singulex) and Quanterix SIMOA [41, 42], alternative and more sensitive HTT detection assays can now also be used by the research community [38–40], which are particularly suited to testing human bio-samples such as CSF or plasma. However, these assays’ throughput remains lower and the resources required to run them higher compared to the MSD platform, which still offers a unique balance between sensitivity and throughput, with assay linearity over a greater concentration range, especially with preclinical samples where multiple tissues need to be tested across a range of species. In addition, an MSD platform-based assay has recently been published for aggregated HTT [25]. TR-FRET based assays for mHTT [43] also allow higher throughput and fewer washing steps, and these assays for mHTT have been reported to be protein conformation-sensitive [44] which could suggest their use in a more biochemical screening effort for molecules that directly affect the conformation of mHTT. In general, we would recommend TR-FRET based assays when using (high throughput) cell culture models, MSD-based assays when using rodent tissue samples, and single molecule counting assays when analyzing biosamples such as human CSF or plasma and animal model CSF and plasma.

Assay transfer and validation across multiple laboratories is crucial to making assays available to the wider research community and also ensuring that data comparison is meaningful and can be aggregated [45, 46]. Method transfer is a distinct process from method validation [47] and is especially important when drug discovery transitions to drug development. Important factors to confirm in the second laboratory are linearity and accuracy (analyte recovery in the intended matrix). A recent review of the cross-laboratory validation of multiple pharmacodynamic biomarker assays highlighted the importance of the availability and qualification of large batches of specialized reagents [48]. Switching to mAbs has enabled us to standardize the assays using large batches of reagents.

All HTT quantification assays reported here have been benchmarked with purified recombinant proteins as a standard, including both large N-terminal fragments (1–573 aa) and full-length (FL; 1–3144 aa) HTT proteins, allowing both quality control of every plate or run and relative HTT quantification when using the recombinant proteins as a standard. One exception will be the mHTT assay as the epitope for MW1 (thought to be (Gln)_~10_) is repeated a number of times in the expanded HTT protein [49], precluding absolute quantification unless the exact same polyglutamine length and context present in the samples is also used as a standard. Consistently, we have observed a different signal over background when comparing large fragment (1–573 aa) and FL (1–3144 aa) HTT as a standard protein, with the latter resulting in approximately one log lower signals. There are a couple of possible explanations for this: folding of FL HTT could be slightly different to that in the large fragment HTT, resulting in less exposure of the N-terminal domain where the antibody epitopes are clustered; or different post-translational modification since the large fragments are purified from insect cells and the FL HTT proteins from human cells. It has for instance been reported that antibody 2166-4C8 is sensitive to phosphorylation [34]. A very similar difference (one log) in signals from N573 versus FL HTT protein was also reported on the Single Molecule Counting Erenna/xPro (Singulex) platform [38], suggesting that this observation is not related to the quantification method itself but inherent to the analyte or the manner the antibodies bind to the epitope in the analyte.

A further indication that large fragment HTT and FL HTT behave differently is indicated by the data generated here on time-course cohorts of mice from a fragment HTT model (R6/2) and a FL HTT model (zQ175DN). As reported previously [16] and replicated with the full mAb assays, we observed a dramatic reduction in soluble human (mutant) exon 1 HTT in R6/2 when measured with either 2B7/MW1 (polyglutamine-dependent) or with 2B7/4C9 (polyglutamine length-independent) (Fig 4). When this is compared with data from the FL Q175 neo- (zQ175DN) model [32, 33], a similar trend (relative to disease progression in these two animal models) is observed when using polyglutamine-dependent assay 2B7/MW1 with a progressive reduction in soluble mHTT in brain hemispheres from 24 weeks onwards (Fig 5A and 5C). The data match the reported increase in aggregated HTT in these mouse models [25], suggesting a shift from soluble mHTT to aggregated mHTT over time. However, the polyglutamine length-independent assay (2B7/4C9) for the same transgenic FL HTT shows very similar levels of soluble mHTT from 12 weeks to one year, replicated in the second cohort (Fig 5D). These data suggest that absolute concentrations of soluble FL mHTT may not significantly change over time, but accessibility to the polyglutamine sequence for the MW1 antibody becomes shielded or the structure of the polyglutamine epitope changes its affinity towards the MW1 antibody. A recent paper however did not observe a discrepancy between the two types of assays in the cortex and striatum of zQ175DN mouse model [24]; furthermore we also found a downward trend of mHTT as measured by 2B7/4C9 in a cohort of zQ175 neo containing mice (which have reported to show a 50% lower expression compared to the zQ175DN mice [32]) It could be possible that changes in mHTT levels in cortex and striatum are masked when analyzing a complete brain hemisphere, although in mouse, cortex and striatum make up a significant percentage of brain volume. A recent cryo-EM HTT structure has suggested that the exon 1 N-terminal domain is extremely flexible compared to the bulk of the protein [50]. It is unlikely that HTT fragmentation would immediately explain this as no cleavage site has yet been reported between the polyglutamine sequence recognized by MW1 and the poly-proline sequence recognized by 4C9. In another FL HTT model (HdhQ150), an increase in insoluble mHTT has been reported over time [51] but, unfortunately, no 4C9-based assay was employed in that study, limiting direct comparison with our data. Degradation by autophagy or proteasome could also be different for the different conformational forms of mHTT; when comparing MW1 and another polyglutamine-specific antibody, 3B5H10, even these closely related assays appeared to recognize pools of mHTT with a different half-life [52]. It is unlikely that cell loss would account for the mHTT signal loss as measured by 2B7/MW1 since the reduction of brain volume in Q175 is relatively subtle in heterozygotes up to 12 months [30]. The discrepancy between the R6/2 fragment HTT model and the Q175 FL HTT model in the time-course of mHTT protein levels suggest that manner of aggregation of mHTT in the aggressive mHTT fragment model may be different from the FL mHTT model, for instance resulting in changes in the accessibility of conformational epitopes from the polyglutamine expansion.

In summary, these fully monoclonal antibody-based HTT quantification assays have been qualified with biologically-relevant pre-clinical samples in two laboratories. With a low LLOD and excellent linearity, these assays are suitable for evaluating candidate therapeutics where reducing HTT and/or mHTT levels are the primary mechanism of action. We would suggest that researchers use the most appropriate reference proteins as standards in these assays, but accept that the quantification will not be absolute, especially when analyzing samples with a different polyglutamine lengths compared to the reference protein using the 2B7/MW1 assay. Using these standards within a single rodent model of HD will allow comparison of data between plates, experiments and between laboratories.

## Supporting information

S1 Fig Purification of recombinant HTT-Q45 (1–573) protein. SDS-PAGE of FLAG affinity and size exclusion purified HTT-Q45 (1–573) protein. M, molecular weight marker (kDa). Ld, sample after FLAG affinity chromatography and loaded onto the size exclusion column. Peak fractions indicate the purity after the size exclusion step.
(EPS)

S2 Fig Purification of human HTT (1–1344) Q17 and Q46 proteins. (A) SDS-PAGE of HTT (1–3144) Q17. Lane 1 = Soluble extract, Lane 2 = Early FLAG flow through fraction, Lane 3 = Late FLAG flow through fraction, Lane 4 = FLAG resin eluate, Lane 5 = 0.5 μg HTT-Q17 1–3144 FLAG-His8 after size exclusion, Lane 6 = 1.5 μg HTT-Q17, 1–3144 FLAG-His8 after size exclusion. (B) SDS-PAGE of HTT (1–3144) Q46. Lane 1 = Soluble extract, Lane 2 = Soluble fraction after filtration, Lane 3 = Early FLAG flow through fraction, Lane 4 = Late FLAG flow through fraction, Lane 5 = FLAG resin eluate, Lane 6 = 0.5 μg HTT-Q17, 1–3144 FLAG-His8, Lane 7 = 1.5 μg HTT-Q17, 1–3144. M, molecular weight marker (kDa).
(EPS)

S3 Fig Characterization of novel rabbit monoclonal antibody CHDI-90002133 (clone 10H14L4) specific for mouse HTT and generated using the ABfinityTM platform (Thermo Fisher). Rabbits were immunized with peptides corresponding to the mouse HTT proline-rich domain (PRD; acetyl-pppQPPQPPPQGQPPPPC-amide). Antibody reactivity and selectivity for mouse HTT was characterized by Western blot using the following samples: recombinant mouse full-length HTT-Q7 (1–3120; “mQ7”) and human HTT-Q23 (1–3144; “hQ23”), and brain lysates from wild type, heterozygous (with only one *Htt* allele encoding the mouse PRD) and homozygous Q175DN (delta-neo) mice (with no mouse *Htt* allele). After denaturation at 70 degrees C, samples were loaded onto a NuPAGE 3–8% Tris-acetate gel (Thermo Fisher). The gel was run at 150 V for 60 minutes. Proteins were transferred from the gel onto Immobilon PVDF membrane using the iBlot 2 Dry Blotting System (Thermo Fisher). After blocking with SEA BLOCK the membranes were probed with primary antibodies CHDI-90002133 (1:500) and mouse anti-GAPDH (1:500). Primary antibody binding was visualized with IR800CW-conjugated goat anti-rabbit and IR680RD-conjugated anti-mouse secondary antibodies (Li-Cor; 1:5,000); imaging was performed with the Odyssey CLx from Li-Cor.
(EPS)

S4 Fig Spike and recovery data for detection of recombinant HTT by ‘polyclonal’ or ‘monoclonal’ MSD assays in mouse brain lysate. Human HTT-Q73 (1–573) was spiked into lysis buffer or different concentrations of wild type mouse brain lysate; mouse full-length HTT was spiked into lysis buffer or brain lysate from homozygous zQ175DN knock-in mice, which have no endogenous mouse *Htt* exon 1 sequence. Data were used for calculation of LLoD shown in Table 1. Some matrix inhibition is noted for all assays, evident as curves in brain lysate being slightly right-shifted compared to curves in lysis buffer. Please note that for assay 2B7/4C9 (polyglutamine-independent human HTT) there is some cross-reactivity with endogenous mouse HTT in the WT brain lysate, causing values above the lysis buffer only curve at the lower concentration range.
(EPS)

S5 Fig Recombinant HTT protein standard curves obtained in two independent laboratories with MSD assays. Standard curve points are indicated in blue; unknown samples in red (corresponding results are shown in Fig 5).
(EPS)

S6 Fig Sequence comparison (mouse / rat / human) of the HTT exon 1 proline-rich domain epitope (green highlight) for mAb2133. Sequences were from GenBank: Human: NP_002102.4; Rat: NP_077333.2; Mouse: NP_034544.1.
(EPS)

S7 Fig Time course analysis of zQ175 (neo containing) mice using either the polyglutamine expanded HTT assay 2B7/MW1 or the polyglutamine length-independent assay 2B7/4C9. (EPS)

S1 Table Overview of recombinant purified HTT proteins used for HTT immunoassays in this paper. Except for HTT-Q45 (1–573) and the three full-length HTT proteins, production of recombinant HTT proteins was previously described in [16].
(EPS)
